# Protocol for a single-centre randomised controlled trial of multimodal periarticular anaesthetic infiltration versus single-agent femoral nerve blockade as analgesia for total knee arthroplasty: Perioperative Analgesia for Knee Arthroplasty (PAKA)

**DOI:** 10.1136/bmjopen-2015-009898

**Published:** 2015-12-21

**Authors:** P D H Wall, A P Sprowson, N Parsons, H Parsons, J Achten, S Balasubramanian, M L Costa

**Affiliations:** 1 Warwick Clinical Trials Unit, Warwick University, Coventry, UK; 2 University Hospitals Coventry and Warwickshire NHS Trust, Coventry, UK; 3 Nuffield Department of Orthopaedics, Rheumatology and Musculoskeletal Science, Oxford University, Oxford, UK

**Keywords:** PAIN MANAGEMENT

## Abstract

**Introduction:**

Total knee arthroplasty (TKA) surgery causes postoperative pain. The use of perioperative injections around the knee containing local anaesthetic, opiates and non-steroidal anti-inflammatory drugs has increased in popularity to manage pain. Theoretical advantages include reduced requirements for analgesia and earlier mobilisation. We propose a single-centre randomised controlled trial of multimodal periarticular anaesthetic infiltration versus femoral nerve anaesthetic blockade as analgesia for TKA. The aim is to determine, in patients undergoing TKA, if there is a difference in patient-reported pain scores on the visual analogue scale (VAS) prior to physiotherapy on day 1 postoperatively between treatment groups.

**Methods and analysis:**

Patients undergoing a primary unilateral TKA at University Hospitals Coventry and Warwickshire Hospitals will be assessed for eligibility. A total of 264 patients will provide 90% power to detect a difference of 12 mm on the VAS on day 1 postoperatively at the 5% level. The trial will use 1:1 randomisation, stratified by mode of anaesthetic. Primary outcome measure will be the VAS for pain prior to physiotherapy on day 1. Secondary outcome measures include VAS on day 2, total use of opiate analgesia up to 48 h, ordinal pain scores up to 40 min after surgery, independent functional knee physiotherapist assessment on days 1 and 2. Oxford knee Scores (OKS), EuroQol (EQ-5D) and Douleur Neuropathic Pain Scores (DN2) will be recorded at baseline, 6 weeks and 12 months. Adverse events will be recorded up to 12 months. Analysis will investigate differences in VAS on day 1 between the two treatment groups on an intention-to-treat basis. Tests will be two-sided and considered to provide evidence for a significant difference if p values are less than 0.05.

**Ethics and dissemination:**

NRES Committee West Midlands, 23 September 2013 (ref: 13/WM/0316). The results will be disseminated via peer-reviewed publications and conference presentations.

**Trial registration numbers:**

ISRCTN 60611146 and EUDRACT Number 2013-002439-10 (protocol code number PAKA-33601-AS117013); Pre-results.

**Strengths and limitations of this study**

- Pragmatic design to facilitate implementation within routine clinical practice.
- Optimised protocol to reduce risk of bias.
- Appropriate sample size calculation and a planned intention-to-treat analysis.
- Lack of blinding among clinicians delivering the intervention.
- Single-centre design.

## Introduction

Total knee arthroplasty (TKA) is the commonest joint replacement, with over 82 000 procedures performed annually in the National Health Service (NHS).^1^ Demand is growing and this, combined with an ageing population, the frequency of TKA and its burden on the NHS increases year on year. During the last decade, there has been increased interest in optimal perioperative care to enhance recovery following TKA. Improvement of analgesia; reduction of surgical stress responses and organ dysfunctions; including nausea, vomiting and ileus; early mobilisation; and oral nutrition have all been examined. Measures to try and improve pain management have been developed including multimodal regimes which theoretically allow functional rehabilitation to be initiated more rapidly postoperatively. TKA generates substantial amounts of postoperative pain, which effects range of movement and ability to mobilise. Good pain relief with minimal physiological disturbance is important for postoperative knee rehabilitation.^2–4^ Epidural analgesia is very effective in pain control but is associated with side effects such as pruritus, urinary retention and motor block.^2^ Epidural analgesia can also occasionally lead to serious complications such as spinal cord ischaemia, vertebral canal haematoma, vertebral canal abscess, infective meningitis, nerve and spinal cord injury, wrong route administration and cardiovascular collapse.^5^

The use of opioid drugs, administered by means of either patient-controlled analgesia or other methods, is another effective method of postoperative pain relief but is often associated with systemic side effects, including nausea and vomiting, respiratory depression, drowsiness, pruritus, reduced gut mobility, and urinary retention.^6^ Drowsiness in particular may delay the patient's postoperative mobilisation. Femoral nerve block, as a single perioperative infiltration or via an indwelling catheter, has been shown to improve postoperative pain control and reduce the use of systemic analgesics and is currently the standard care for perioperative analgesia.^7^ The key advantage of the technique is that it avoids the systemic effects associated with both epidural and opioid analgesics. However, it may be associated with complications such as vascular puncture, nerve damage, infection and diminished muscle control.^8^ The inhibition of the quadriceps muscle group can delay postoperative mobilisation.^9^ Since the posterior capsule of the knee joint is innervated by the branches of the sciatic nerve rather than the femoral, femoral nerve blockade may also result in incomplete pain relief. Femoral nerve block is currently the standard perioperative analgesia for TKR surgery among anaesthetists working within the NHS.

Recently the use of intraoperative, periarticular infiltration of multimodal analgesics has gained in popularity. Periarticular infiltration has the advantage of delivering drugs directly to the sources of pain, thereby avoiding systemic side effects.^10^ The concept of multimodal analgesia refers to the simultaneous administration of multiple anaesthetic agents, such as local anaesthetics, opiates and non-steroids anti-inflammatory drugs (NSAIDs). To produce optimal pain relief combined with the lowest incidence of side effects, a multimodal pain therapy is essential.^5^ This technique of analgesia was developed specifically to avoid sedation and facilitate rapid physiological recovery after lower limb arthroplasty in order to enable early mobilisation and discharge.^9–13^ In contrast to femoral nerve blockade, periarticular infiltration does not inhibit quadriceps function and also reaches the posterior capsule of the knee joint. Published studies suggest that periarticular infiltration may reduce requirements for postoperative analgesia, lead to earlier mobilisation and discharge from hospital. However, the number of published randomised controlled trials involving TKA is small and all are underpowered and lack statistical rigour. An initial pilot study comparing femoral nerve block and multimodal periarticular infiltration has already been completed in order to help plan and design a full trial with the following null hypothesis.^14^

Postoperative pain following primary TKA does not differ between multimodal periarticular knee infiltration with levobupivacaine 150 mg, morphine 10 mg and ketorolac 30 mg diluted in 0.9% saline to make a volume 100 mL (0.5 mL 1:1000 epinephrine) and the single-agent femoral nerve blockade.

These two comparators have been chosen for comparison because femoral nerve block is the current standard care for perioperative analgesia for TKR surgery and multimodal periarticular infiltration represents a new but now established alternative.

## Objectives

The primary objective of this full trial is to quantify and draw inferences on the efficacy between treatment groups based on observed differences as shown by a validated, patient-reported 100 mm visual analogue pain score, prephysiotherapy on the first postoperative day, collected by an independent physiotherapist. This is the most important outcome as pain at the time when the patient is first starting to walk and use their new knee replacement will determine the ability of the patient to mobilise. Early mobilisation is associated with improved functional outcomes and a reduced risk of complications.^15^

The secondary objectives of the study are to quantify and draw inferences on the efficacy of the treatment groups based on observed differences as shown by:

1. Visual analogue scale (VAS) after physiotherapy on the first postoperative day and before and after physiotherapy on the second postoperative day.
2. The total use of opiate analgesia up to 24 and 48 h after the operation.
3. Ordinal pain score (routinely collected up to 40 min after surgery).
4. Independent routine functional physiotherapist assessment on days 1 and 2 postoperatively assessing: straight leg raise (SLR), knee range of movement, Timed Up and Go (TUG), bed transfers and distance mobilised.
5. Oxford Knee Score (OKS) collected preoperatively and 6 weeks postoperatively.
6. EuroQol (EQ-5D-5L) Score collected preoperatively and 6 weeks postoperatively.
7. Douleur Neuropathic Pain Scores (DN2/seven-item DN4) collected preoperatively and 6 weeks and 12 months postoperatively.^16^ ^17^
8. The number and type of adverse events (AEs) up to 12 months postoperatively.

## Methods and analysis

The protocol (V.5.0 dated 7 October 2015) was prepared in accordance with the Standard Protocol Items: Recommendations for Interventional Trials (SPIRIT) guidelines.^18^ This study is jointly sponsored by the University of Warwick and University Hospitals Coventry and Warwickshire NHS Trust. The trial will be carried out in accordance with the Medicines for Human use (Clinical Trials) Regulations 2004 (SI 2004/1031), amended regulations (SI 2006/1928) and the International Conference on Harmonisation Good Clinical Practice (ICH-GCP); all collaborators will be trained in GCP, and in accordance with this protocol. This trial will be reported in line with the Consolidated Standards of Reporting Trials (CONSORT) statement.

A single-centre two-arm parallel group superiority type trial design will be completed. All patients undergoing an elective primary unilateral TKA under the care of an orthopaedic consultant at University Hospitals Coventry and Warwickshire NHS Trust are potentially eligible for entry to the trial. However, patients with any of the following will not be eligible:

1. Concomitant medical or psychiatric problems which, in the opinion of the investigator, would prevent completion of treatment or follow-up.
2. Preoperative history of neurological abnormality in the ipsilateral leg, for example, history of stoke, neurogenic pain or previous nerve pain.
3. Specific contraindication to the analgesic agents used:
  - *Morphine*
    i. Hypersensitivity reaction.
  - *Ketorolac*
    i. Active or previous peptic ulcer;
    ii. History of upper gastrointestinal bleeding or perforation, related to previous NSAID therapy;
    iii. Haemorrhagic diatheses, including coagulation disorders;
    iv. Hypersensitivity to ketorolac, trometamol or other NSAIDs;
    v. Moderate or severe renal impairment (serum creatinine >160 μmol/L).
  - *Levobupivacaine*
    i. Known hypersensitivity to levobupivacaine, local anaesthetics of the amide type or any of the excipients;
    ii. Uncontrolled angina;
    iii. Second or third degree heart block.
4. Participation in a clinical trial of an investigational medicinal product (IMP) in the past 90 days.
5. Previous entry in the present trial.
6. Evidence that the patient would be unable to adhere to trial procedures.

Patients fulfilling the eligibility criteria will be identified by consultants and research associates in outpatient clinics. In order to ensure all eligible patients are approached for recruitment patients already on the waiting list (as identified via hospital operative planning software, Opera) for a unilateral TKA will be screened and may be contacted during their preoperative assessment at the hospital, which normally occurs a few weeks before surgery. All appropriate patients will be approached as per ICH-GCP guidelines. Patients will be recruited by trained research associates who will help to present the trial and interventions in a consistent and unbiased manner. Recruitment by trained research associates will also be a mechanism to help ensure optimum participant enrolment.^19^ Patients will only be given ‘Letters of Invitation’ if, in the opinion of the research associate, there has been an adequate verbal introduction to the trial. Patients will be given adequate time to consider their participation in order to ensure informed consent to participate in the trial. Signed and dated informed consent will be obtained by medically trained personnel as per trust protocol for Clinical Trial Investigation of a Medicinal Product (CTIMP) study. In the event that any further information becomes available, which may influence the patient's willingness to continue in the trial, the trial team will contact the participant. The participant's general practitioner (GP) will be informed by letter that the patient is taking/has taken part in this clinical trial. A participant may deny the research team permission to inform the GP of their trial involvement by not initialling the appropriate box on the consent form. Prerandomisation eligibility checks will be carried out to ensure that a patient fit the eligibility criteria and is not randomised in error. Inclusion of a patient in the trial will be flagged on their clinical notes by means of a trial sticker.

## Randomisation

Allocation of trial treatments will be provided through a distal randomisation service. Randomisation will be a 1:1 allocation using a computer-generated randomisation schedule stratified by anaesthetic type—general or spinal block using permuted blocks of random sizes. The block sizes will not be disclosed, to ensure concealment. To ensure allocation concealment, the mechanism of contact being used is via a telephone and has a stringent procedure to ensure enrolment before randomisation. Randomisation via telephone will be undertaken by a trained member of the theatre team present on the day of surgery. They will then inform the rest of the theatre team (excluding the participant) of the treatment allocation.

## Sample size

The primary outcome measure for this study is pain on day 1 postoperatively, assessed using a 100 mm VAS. Pilot data (n=46) were used in a power analysis to estimate the sample size required for a two-arm parallel group RCT. Based on the available literature, a change in the VAS of 12 mm (95% CI 9 to 15) is clinically meaningful; thus, these calculations assume the minimum clinical important difference (MCID) to be approximately 12 mm.^20^ The observed SD from the pilot study was 30 mm, giving a standardised effect size (MCID/SD) of 0.4, a moderate value, and of the appropriate order of magnitude for a pragmatic study of this type. Hence, to power a trial to test the null hypothesis of equality of the treatments, assuming approximate normality for the VAS, would require 132 patients in each treatment arm or 264 in total—assuming 90% power, 5% significance, a SD of 3 cm and an MCID of 12 mm. Given that the majority of data collection will occur during participant hospital stay, with the exception of the patient-reported outcome measures, we anticipate loss of follow-up data will be minimal (<5%). The sample size for this trial corresponds to effects observed in previous similar studies.^20^ ^21^ These studies demonstrated effects on a 100 mm VAS (our primary outcome measure) and on participant consumption of ‘as required’ analgesia with 20–25 patients per experimental group.

Recent audit within the department indicated there are approximately 50 elective primary unilateral total knee replacements carried out per month, of whom over half would be eligible for this trial. Although not all patients will want to take part, our previous experience in trials of perioperative adjuncts to surgery has shown high levels of patient recruitment (80–100%) with only 7% declining the pilot study. Therefore, we believe 11 patients per month to be a realistic recruitment figure. At this rate, the entire study sample can be recruited within 24 months. However, if recruitment rate is not as high as anticipated, a sample size of 200 patients will still be adequate to identify any difference between groups with 80% power.

Participants may withdraw from the trial treatment and/or the whole trial at any time without prejudice. Unless a participant explicitly withdraws their consent, they should be followed-up and data collected as per the protocol until the end of the trial.

Should a participant withdraw from the trial, they would continue to be treated as per normal routine postoperative management, follow-up and clinical practice. The data collected up until the point of withdrawal would be used for analysis at the end of the trial. Participants may be withdrawn from the trial at the discretion of the investigator and/or the Trial Steering Committee (TSC) due to safety concerns.

## Blinding

Patients will be blind to the intervention to which they are allocated, as femoral nerve blocks will be done after sedation and or anaesthetic. All interventions will be conducted within a sterile zone with drapes which will physically prevent patients seeing which intervention they receive. Owing to the nature of the study, it is not possible for the surgeon and anaesthetist delivering the interventions to be blinded to the treatment options. Outcome data will be collected by a research associate and an independent clinical physiotherapist who are blinded to the treatment allocation. Furthermore, the trial statistician will be blinded to the treatment allocations throughout.

## Interventions

In this pragmatic trial, patients will undergo routine elective primary unilateral TKA using the standard technique of the anaesthetist and the operating surgeon. In addition, the patient will receive one of the following perioperative analgesic interventions:

1. Femoral nerve block

Under aseptic conditions, the femoral artery will be palpated immediately below the inguinal ligament and nerve stimulation and or ultrasound will be used to identify the femoral nerve just lateral to the artery. Once the femoral nerve has been identified, the block may be performed in the routine manner (15, 16) using 30 mL (75 mg) of levobupivacaine hydrochloride 0.25%.

1. Intraoperative periarticular injection

The periarticular infiltration of multimodal agents will involve the preparation of two 50 mL syringes each containing 30 mL (75 mg) of levobupivacaine hydrochloride 0.25% injection, 0.5 mL (5 mg) morphine sulfate injection, 0.5 mL (15 mg) ketorolac trometamol injection and 0.25 mL of 1:1000 epinephrine then diluted with 0.9% saline to make a mixture containing a total volume of 50 mL. Epinephrine is added to the mixture to reduce blood loss after the operation. Each syringe will be prepared for immediate use and not stored. Fifty millilitre of the mixture will be injected into the posterior, medical and lateral soft tissues just prior to implantation of the TKR components. Care will be taken to avoid excessive infiltration in the area of the common peroneal nerve. Then, while the cement is curing, the anterior soft tissue including the quadriceps mechanism, the retinacular tissues and the subcuticular tissues will be infiltrated with the remaining 50 mL of periarticular injection.^13^ Following wound closure, the tourniquet will be released and the ‘tourniquet-down time’ noted on the trial documentation.

The allocated intervention will be discontinued if there is evidence of an immediate serious adverse reaction such as anaphylaxis. Once randomised if a participant specifically requested that the intervention was discontinued or modified this would be honoured provided valid consent was obtained; however, as both interventions are delivered perioperatively such a scenario is extremely unlikely.

A routine preoperative, perioperative and postoperative analgesic medicines regimen will be used for all of the participants following hospital guidelines for TKR surgery:

*Premedication (before surgery)*

- 1. Gabapentin 300 mg (100 mg if older than 70 years or chronic kidney disease (CKD) stage 3).

*Perioperatively*

- 1. Spinal: 2 mL of 0.5% heavy bupivacaine or 2 mL of 0.5% isobaric levobupivacaine (chirocaine).
- 2. Sedate with target-controlled infusions of propofol or general anaesthetic if needed.
- 3. If unable to do a spinal, use intravenous morphine 0.1–0.2 mg/kg intraoperatively.
- 4. Paracetamol: 1 g intravenous.

*Postoperatively*

- 1. Paracetamol 1 g four times a day;
- 2. Diclofenac 50 mg or ibuprofen 400 mg three times a day if no contraindications and to be started 8 h postoperatively;
- 3. Gabapentin 300 or 100 mg three times a day for 5 days (lower dose for the over 70 s or CKD stage 3);
- 4. Morphine sulphate tablets (MST) 20 mg twice daily for 5 days or until needed first dose in recovery before spinal wears off;
- 5. Oramorph 10 or 20 mg (maximum hourly) as required.

*On discharge*

- 1. MST 10/20 mg twice daily (to cover 5 postoperative days);
- 2. Gabapentin 300 mg three times a day (100 mg three times a day for over 70 s; to cover 5 days postoperative);
- 3. Paracetomol 1 g four times a day;
- 4. Ibuprofen/diclofenac 400 mg/50 mg three times a day.

All postoperative analgesia taken by the participants, both regular and as required (prn), will be recorded. All of the participants will follow the standard university hospitals coventry and warwickshire (UHCW) postoperative rehabilitation protocol under the supervision of a physiotherapist. This involves immediate full weight bearing with the use of crutches, no restriction in flexion and the regular use of a cryocuff for cold therapy.

The fidelity with which both interventions are delivered will be captured by regular audits against the standards described. The results will be relayed to those delivering the intervention in order to improve and/or maintain ongoing protocol compliance.

## Outcome assessments and time points

We will use techniques common in long-term cohort studies to ensure minimum loss to follow-up, such as collection of multiple contact addresses and telephone numbers, mobile telephone numbers and email addresses. Trial outcome assessment time points are shown in Table 1.

**Table 1:** Trial outcome variables, analysis metric, aggregation method and time points

			Time point				
Outcome variable	Analysis metric	Aggregation	Baseline	Day 1 postoperative	Day 2 postoperative	Week 6 postoperative	Month 12 postoperative
VAS (0–100 mm)	Final value	Mean		× (before and after physiotherapy)	× (before and after physiotherapy)		
4-point pain scale	Final value	Proportion and type		×	×		
Total use of opiate analgesia (mg)	Final value	Mean		×	×		
Straight leg raise (yes/no)	Final value	Proportion		×	×		
Knee ROM (degrees)	Final value	Mean		×	×		
Timed Up and Go (impaired mobility yes/no)	Final value	Proportion		×	×		
Bed transfers (4-point ordinal scale)	Final value	Proportion and type		×	×		
Distance mobilised (metres)	Final value	Mean		×	×		
OKS	Final value	Mean	×			×	×
EQ-5D-5L	Final value	Mean	×			×	×
DN2 (7-item DN4 ordinal scale)	Final value	Proportion	×			×	×
Adverse events	Final value	Proportion and type		×	×	×	×

DN, Douleur Neuropathic Pain Score; EQ-5D-5L, EuroQol; OKS, Oxford Knee Score; ROM, range of motion; VAS, visual analogue scale.

Our primary outcome measure will use the well-established 100 mm VAS reported by the participant prior to physiotherapy on first day postoperatively, as this is when the patient would be expected to get out of bed and mobilise the knee after their surgery. The mean VAS score will be reported for both treatment groups. A further VAS measurement will be performed before physiotherapy on the second day. This will allow us to define the analgesic effect following mobilisation. Any failure to mobilise and the reason for failure will be recorded from the patient's physiotherapy record. Additional routine standard of care pain score data will be collected during the patient's hospital admission. The pain score is a four-point ordinal scale. The pain data will be reviewed by the research associate and entered onto an anonymised participant data sheet. Early knee function will be assessed by an independent physiotherapist in both groups of patients using four basic methods:

1. SLR: with patient supine the participant is to attempt (unaided) to flex at the hip with knee locked in extension to raise their operated-side ankle off the bed. If the participant is able to raise ankle at least 5 cm off bed the bed, they are deemed to be able to SLR. The proportion of participants able to SLR in each group will be reported.
2. Knee range of movement: the patient's own active knee range of motion (ROM) to both extension and flexion will be measured in degrees. The mean knee ROM in each group will be reported.
3. The participant is assessed in their ability to transfer from bed to chair: (1) independently, (2) with assistance of one, (3) with assistance of two and (4) unable to mobilise. The proportion of participants in each group will be reported for this ordinal scale.
4. TUG is a test of functional mobility. It uses the time that a person takes to rise from a chair, walk 3 m, turn around, walk back to the chair and sit down. During the test, the patient is expected to wear their regular footwear and use any mobility aids that they would normally require. A time of >20 s indicates impaired mobility. The proportion of participants with impaired mobility in each group will be reported.

At 48 h postoperatively, participant drug charts and anaesthetic charts will be reviewed by the research associate. Opiate analgesia used will be converted to ‘morphine equivalent dose’ (see table 2).^22^ Total morphine equivalent dose used up to 24 and 48 h postoperatively will be recorded for each participant in milligrams and the mean dose reported for each treatment group. The total dose of paracetamol and/or NSAIDS will also be reported.

**Table 2:** Opiate analgesia converted to morphine equivalent dose

Opiate analgesia	Route	Typical dose	Total 24 h dose	Equivalent morphine 24 h dose	Four hourly oral morphine dose	Relative potency to oral morphine (24 h)
Codeine^22^	Oral	60 mg four times a day	240 mg	24 mg	4 mg	0.1
Dihydrocodeine^22^	Oral	60 mg four times a day	240 mg	24 mg	4 mg	0.1
Tramadol^22^	Oral	50 mg four times a day	240 mg	40 mg	6.6 mg	0.2

At 6 weeks and 12 months postoperatively, all participants will asked to complete some questionnaires either at their routine clinical follow-up appointment or via post. The questionnaires will ask participants to complete three validated outcome scores:

1. OKS will assess participant's perceived function following their procedure. This is a validated self-administered osteoarthritis outcome measure and should only require 10 min to complete.^23^ The mean final value for OKS will be reported for both treatment groups at 6 weeks and 12 months.
2. EQ-5D-5L is a validated measure of health-related quality of life, consisting of a five-dimension health status classification system and a separate VAS.^24^ ^25^ The mean final value for EQ-5D-5L will be reported for both treatment groups at 6 weeks and 12 months.
3. DN2/seven-item DN4, a validated screening tool for neuropathic pain consisting of two questions.^16^ ^17^ The proportion of participants with evidence of neuropathic pain in each group will be reported.

All AEs will be recorded up to 12 months after surgery*.* An AE is defined as any untoward medical occurrence in a participant and which does not necessarily have a causal relationship with the treatment. A serious adverse event (SAE) is an AE that fulfils one or more of the following criteria:

1. Results in death;
2. Is immediately life-threatening;
3. Requires hospitalisation or prolongation of existing hospitalisation;
4. Results in persistent or significant disability or incapacity;
5. Is a congenital abnormality or birth defect;
6. Is an important medical condition.

Suspected unexpected serious adverse reactions are SAEs that are unexpected, that is, their nature or severity is not consistent with the summary of product characteristics, and are considered to be caused by one or more of the trial medicinal interventions.

The following (serious) AEs will be expected and therefore will not need immediate reporting to the trial office: chest infection, urinary tract infection, myocardial infarction, stroke, superficial surgical site infection, deep surgical site infection, bleeding, removal/revision of metalwork, deep vein thrombosis (DVT)/Pulmonary Embolism (PE), damage to nerves in the surgical area. The total number, type (with proportions) of AEs will be reported for both groups.

## Data management

The case report forms will be designed by the trial coordinator in conjunction with the trial management team. All electronic patient-identifiable information will be held on a secure, password-protected database accessible only to essential personnel. Paper forms with patient-identifiable information will be held in secure, locked filing cabinets within a restricted area of the Clinical Sciences Building at University Hospitals Coventry and Warwickshire. Participants will be identified by a code number only. Direct access to source data/documents will be required for trial-related monitoring. All paper and electronic data will be retained for at least 5 years after completion of the trial.

## Statistical analysis plan

Standard statistical summaries (eg, means and variances, medians and ranges or proportions dependent on the distribution of the outcome) and graphical plots showing correlations will be presented for the primary outcome measure and all secondary outcome measures. Baseline data will be summarised to check comparability between treatment arms, and to highlight any characteristic differences between those individuals in the study, those ineligible and those eligible but withholding consent.

The main analysis will investigate differences in the primary outcome measure, the VAS pain score prephysiotherapy on the first day postoperatively, between the two treatment groups (single injection femoral nerve block and multimodal periarticular injection) on an intention-to-treat basis. Initial analysis will investigate differences in pain score measurements on an intention-to-treat basis using a t test based on an assumed normal distribution for the primary outcome (VAS pain score). Tests will be two-sided and considered to provide evidence for a significant difference if p values are less than 0.05 (5% significance level). Estimates of treatment effects will be presented with 95% CIs. The simple t test will be augmented with a linear regression analysis that adjusts for expected confounders of age and gender. Adjusted and unadjusted analyses will be presented together with diagnostics that assess the modelling assumptions (eg, quantile-quantile plots). Subsidiary analyses will also test for differences at intermediate times and more generally across all times using a repeated-measures approach (eg, generalised estimating equations). For secondary outcome measures that can be assumed to be approximately normally distributed (eg, OKS, EQ-5D), data will be analysed in a similar manner to VAS pain scores. However, routinely collected pain scores, measured on a four-point ordinal score scale, will be analysed using the proportional odds model and the time course modelled using appropriate methods (eg, repolr). Counts of AEs will be compared between groups using χ^2^ tests.

Inevitably some data may not be available due to voluntary withdrawal of patients, lack of completion of individual data items or general loss to follow-up. Where possible, the reasons for missing data will be ascertained and reported. Although missing data are not expected to be a problem for this study, the nature and pattern of the missing data will be carefully considered including in particular whether data can be treated as missing completely at random. If judged appropriate, missing data will be imputed using the multiple imputation facilities (eg, mice in R). Any resulting imputed data sets will be analysed and reported, together with appropriate sensitivity analyses. Any imputation methods used for scores and other derived variables will be carefully considered and justified. Reasons for ineligibility, non-compliance, withdrawal or other protocol violations will be stated and any patterns summarised. More formal analysis, for example, using logistic regression with ‘protocol violation’ as a response, may also be appropriate and aid interpretation.

The main analyses will be conducted using the software package R (http://www.r-project.org/), with some additional analyses in SPSS if this proves necessary. The primary focus of the statistical analysis will be the comparison of the two treatment groups, and this will be reflected in the analysis which will be reported together with appropriate diagnostic plots that check the underlying model assumptions.

## Trial organisation, regulation and oversight

All issues pertaining to the management of the trial will be coordinated by a trial management group (TMG). The TMG comprises the chief investigator, trial manager, coinvestigators, trial statistician and the hospital site (UHCW) principal investigator.

The Data Monitoring Committee (DMC) comprises an independent chair with relevant experience in trial statistics, the trial statistician and the trial manager. The main roles of the DMC will be to review/approve the Statistical Analysis Plan, and to review trial progress, interim data and safety aspects of the study.

The TSC comprises an independent chair, chief investigator, trial manager, coinvestigator, statistician, an independent public representative and sponsor representative. The remit of the TSC is to:

- Monitor and supervise the progress of the trial towards its interim and overall objectives.
- Review at regular intervals relevant information from other sources.
- Consider the recommendations of the DMC.
- Inform the funding body on the progress of the trial.

Any proposed changes to the protocol will first be reviewed by the TSC and if approved then submitted for independent review and approval by the trial sponsor and local research ethics committee. Substantive amendments defined as changes that may affect the safety of trial participants or the scientific validity, scope or ethical rigour of the trial will also be communicated to the trial registries and funding body. All approved protocols will be marked by a version number and date.

The trial is registered with the International Standard Randomised Controlled Trial Number Register, the Medicine and Healthcare Products Regulatory Agency (MHRA) UK and EudraCT. The study will conform to regulations for a CTIMP. The blinding will only be broken for clinical management purposes. In exceptional circumstances beyond this agreement will be sought from the chief investigator and statistician before the blinding is broken.

For this trial, levobupivacaine hydrochloride 0.25% injection, morphine sulfate injection, ketorolac trometamol injection, 1:1000 epinephrine injection and sodium chloride 0.9% injection used perioperatively are being used as IMPs. All IMPs will be taken from commercially available stock, and drug accountability logs for IMPs will be maintained by the chief investigator and those individuals with designated responsibilities. Accountability logs will record the manufacturer, batch number, expiry dates and the patient's trial number, in order to maintain traceability of the stock issued within the trial. All records will be maintained in accordance with current GCP and in line with the Medicines for Human Use (Clinical Trials) Regulations 2004.

The allocated recruitment period for the trial is 24 months. Recruitment began in December 2013 and is due to finish in December 2015. Once recruited participants are randomised to a treatment allocation within 3 months and then followed up for 12 months. It is anticipated that the trial will be finished by March 2017. The trial has been funded for participant follow-up to 6 weeks after surgery and a study report to the funders is anticipated by May 2016.

## Ethics and dissemination

The definitions of the EU Directive 2001/20/EC article 2 based on ICH-GCP apply in this trial protocol. Both investigators and sponsors will follow specific procedures when notifying and reporting AEs/adverse reactions in this trial. SAEs that are not listed as expected will be considered to be related or potentially related to the administration of the IMP. Expectedness will be determined by the investigators using the information within the products SPC. SAEs that are deemed to be unexpected and related to the trial will be notified to the main research ethics committee, MHRA and trial sponsor within 15 days for a non-fatal or non-life-threatening event and within 7 days for a fatal or life-threatening event. All participants experiencing SAEs will be followed-up as per protocol at the end of the trial and causality of SAEs assessed.

Participant in the study are covered by indemnity for negligent harm through the standard NHS indemnity arrangements. The University of Warwick has insurance to cover for non-negligent harm associated with the protocol. The liability of the manufacturer of medicinal products being administered is strictly limited to those claims arising from faulty manufacturing of the product.

The results of the trial will be disseminated via patient information material prepared in collaboration with NHS Choices. All key findings from the trial will be presented at national and international conferences such as the British Orthopaedic Association (BOA) and British Association of Specialist Knee Surgeons (BASK), and we aim to publish the results in at least one major peer-reviewed publication.

## Funding and sponsorship

This study protocol presents independent research funded by the National Institute for Health Research (NIHR) under the Research for Patient Benefit Scheme: PB-PG-0212-27098. The views expressed are those of the authors and not necessarily those of the NHS, the NIHR or the Department of Health.

The study is jointly sponsored by the University of Warwick (Mrs Jane Prewitt) and University Hospitals Coventry and Warwickshire NHS Trust (Mrs Ceri Jones). The trial sponsors provide ultimate approval of all new versions of the protocol before they become live. Both the funders and sponsors are required to provide final approval before publication of any study material.
